# Meal patterns, including intermittent fasting – a scoping review for Nordic Nutrition Recommendations 2023

**DOI:** 10.29219/fnr.v68.10505

**Published:** 2024-02-06

**Authors:** Mette Svendsen, Heléne Bertéus Forslund

**Affiliations:** 1 Department of Endocrinology, Obesity and Preventive Medicine, Oslo University Hospital, Oslo, Norway; 2 Department of Internal Medicine and Clinical Nutrition, Sahlgrenska Academy, University of Gothenburg, Gothenburg, Sweden

**Keywords:** meal patterns, eating frequency, snacking, meal timing, intermittent fasting, dietary recommendations

## Abstract

**Introduction:**

‘Meal patterns’ refers to eating frequency, snacking, regularity, and timing. Here also including intermittent fasting. The effect of meal patterns on health is inconsistent and when updating the Nordic Nutrition Recommendations 2023 (NNR2023), summarizing the evidence is important.

**Aims:**

To describe the evidence for the role of meal patterns on bodyweight, body composition, and cardiovascular risk factors (i.e. blood pressure and lipid- and glukose metabolism) in healthy people living with normal weight, overweight, or obesity in all age groups.

**Methods:**

An initial search in PubMed found 481 reviews, of which 104 were identified based on titles. Of these, 47 were excluded based on title and abstracts. Of the remaining 57 reviews, 16 were included reporting search terms and inclusion/exclusion criteria. In addition, 8 reviews from reference list or known by authors were included. In total, 24 reviews were relevant. Cochrane Library was searched with no results.

**Results:**

All reviews were rated low or critically low (AMSTAR 2). No consistent findings on eating frequency and body weight or composition were found in children/adolescents or adults. In snacking, mixed results were found, although among adults, some consistent results showed positive associations between snacking and body weight. In regularity, breakfast skipping showed mixed results in children/adolescents on body weight and composition. Among adults, randomized controlled trials on breakfast skipping showed a minor impact on improved weight loss. In prospective studies on timing, lower energy intake during late afternoon/evening was related to less body weight. Intermittent fasting reduced body weight but was not superior to continuous energy restrictions. Cardiovascular risk factors were assessed in a minority of the reviews, and despite some beneficial effects, the evidence was limited.

**Conclusion:**

Given the overall low to critically low quality of the reviews, the evidence is limited and inconclusive. No consistent results providing evidence for setting recommendations for meal patterns were shown. In this regard, meal patterns may vary within the context of an energy balanced and nutritionally adequate diet.

## Popular scientific summary

- The term meal patterns refers to eating frequency, snacking, regularity, and timing of meals.
- Meal patterns in the Nordic countries can be described as regular, and most people eat breakfast.
- Snacking may be associated with higher body weight in adults, while lower late afternoon/evening energy intake has been associated with lower body weight or weight loss.
- Intermittent fasting has been shown to reduce body weight similar to continuous energy restriction.
- No causal relationship between meal patterns including intermittent fasting and health related outcomes has been established.

Meal patterns refers to eating frequency or occasions, snacking, timing, and regularity. Regularity may include time between meals, day-to-day variation, or skipping meals. Meal patterns varies between individuals according to age, culture, and traditions. For instance, meal patterns reported from the European Prospective Investigation into Cancer and Nutrition (EPIC) calibration study showed a north-south difference in frequencies of eating occasions and timing. The participants from the Nordic and Central Europe reported more eating occasions compared to southern Europe. Europeans in the north reported about 1/3 of total energy intake from snacking. Furthermore, northern Europe had a higher proportion of energy eaten later in the day compared to the Mediterranean participants, although the latter having later timing of meals (1, 2). However, no clear associations between meal timing and body mass index (BMI), lipids, or type 2 diabetes mellitus were found (2).

The research on meal patterns in relation to health outcomes has shown divergent results. One reason is the lack of consensus about the definitions of eating occasions. Numerous definitions have been proposed by investigators, although the terms ‘meals’ and ‘snacks or snacking’ are commonly used to describe eating occasions (3, 4). ‘Meals’ generally refers to main meals (breakfast, lunch, and dinner) and ‘snacks or snacking’ to in-between meal eating or drinking. Whether only drinks with or without energy are included or not in eating occasions or snacks varies among studies (3, 4). In addition, the distinction between eating occasions can be set by the researcher or by the respondents themselves. This makes the interpretations related to outcomes even more difficult.

Another reason for divergent results in meal pattern research is methodological problems, such as various classification of eating occasions (i.e. cultural, time, preparation, energy, or nutrient content) and different time space when separating eating occasions, as well as underreporting. For example, Murakami and Livingstone showed that the association between the number of eating occasions and BMI and waist circumference changed by different definitions. By using a definition taking energy content of each eating occasion into account as well as under-reporting, inverse associations changed to positive associations (5). Hence, using different time space when separating eating occasions is also crucial.

Meal patterns may have an effect on energy balance, that is energy intake and energy expenditure. Having a high or a low eating frequency can alter energy intake as well as appetite or satiety. However, eating frequency has a minor impact on energy expenditure under iso-caloric conditions. In this regard, meal patterns including timing may have an impact on energy intake, homeostatic (6), and hedonic (7) body weight regulation as well as lipid and glucose metabolism (6, 7) in the fasting and post-prandial state and by that increase risk for cardiovascular disease. In addition, internal factors such as circadian rhythms may also have an impact on eating patterns and health-related outcomes.

Intermittent fasting has been included in the present scoping review as a result of a public hearing process for the Nordic Nutrition Recommendations 2023 (NNR2023) project (8). Fasting has traditionally been used for religious purposes and for general health reasons (9). However, the increased popularity of intermittent fasting during later years is mostly related to the effect on weight loss. Different variants of intermittent fasting exist (Table 1), although the common strategy is to restrict eating in specific periods during the day or week. There is scarce knowledge about the effects of intermittent fasting compared to continuous energy restrictions when similar energy restrictions are compared.

**Table 1:** Variants of intermittent fasting

Strategy	Description
Alternate day fasting	Alternating every other day either eating as usual or fasting (≤25% of energy needs or eating only 400–600 kcal or no energy-containing foods or only drinking).
Intermittent energy/calorie restrictions	Eating as usual 5 days a week and fasting (i.e. 400–600 kcal) for 2 non-consecutive days per week
Time-restricted feeding/eating	Eating as usual within specific timeframe (i.e. 4–10 h) and then fasting for remaining hours of the day (14–20 h).
Periodic fasting/whole day fasting	Fasting for up to 24 h once or twice a week with eating as usual the other days.
Religious fasting	Variety of fasting strategies according to religious rules

The aim of this scoping review was to describe the evidence for the role of meal patterns (eating frequencies, snacking, breakfast skipping, timing, and intermittent fasting) on body weight, body composition, and cardiovascular risk factors (i.e. blood pressure and lipid- and glucose metabolism) in healthy people living with normal weight, overweight, or obesity in all age groups (Box 1).

Box 1 Background papers for Nordic Nutrition Recommendations 2023

- This paper is one of many scoping reviews commissioned as part of the Nordic Nutrition Recommendations 2023 (NNR2023) project (8)
- The papers are included in the extended NNR2023 report, but, for transparency, these scoping reviews are also published in Food & Nutrition Research
- The scoping reviews have been peer reviewed by independent experts in the research field according to the standard procedures of the journal
- The scoping reviews have also been subjected to public consultations (see report to be published by the NNR2023 project)
- The NNR2023 committee has served as the editorial board
- While these papers are a main fundament, the NNR2023 committee has the sole responsibility for setting dietary reference values in the NNR2023 project

## Methods

After an initial search conducted by the NNR2023 Committee, we expanded the search terms and otherwise followed the methodology published previously (10).

PubMed was searched on 07 May 2021 with the following search terms:

“meal pattern*”[Title/Abstract] OR “meal frequenc*”[Title/Abstract] OR “eating frequenc*”[Title/Abstract] OR “meal tim*”[Title] OR “snack*”[ Title/Abstract] OR “meal skipping”[ Title/Abstract] OR “irregular meals” [Title/Abstract] OR “regular meals” [Title/Abstract] OR “eating occasions” [Title/Abstract] OR “breakfast skipping” [Title/Abstract] OR “eating pattern*”[Title/Abstract] OR “nibbling” [Title/Abstract] OR “Chrono-nutrition” [Title/Abstract] OR “circadian rhythm”[ Title/Abstract] OR “Intermittent fasting”[ Title/Abstract] OR “intermittent energy restriction”[ Title/Abstract] OR “alternate day fasting”[ Title/Abstract] OR “time restricted feeding”[ Title/Abstract] AND “diet” [Title/Abstract] AND (“2011”[PDAT] : “3000”[PDAT]) AND Humans[Filter] AND Review[Publication Type]

The initial search identified 481 publications. Both authors read titles, of which 104 reviews were identified. Of these, 47 were excluded based on title and abstracts. Of the remaining 57 reviews, 16 were included reporting search terms and inclusion/exclusion criteria. In all, 41 reviews were excluded as this information was not provided, or outcomes were irrelevant. Publications conducted with normal weight, overweight, and obesity were included, whereas studies in special groups (i.e. patients, religious groups, and shift workers) were excluded. In the updated search on 01 October 2021, six publications were found, but no further studies were included because lack of information about search methods or irrelevant outcomes. In addition, four publications from reference lists and four publications known by authors were included. In total, 24 reviews were relevant for setting guidelines. These were extracted according to data on first author, number of publications, study design, population, intervention/exposure, comparator, dietary method (if relevant), outcomes, and AMSTAR 2 grading in summary tables. When differences were discovered, we discussed and agreed upon the final tables.

Cochrane Library was searched on 31 December 2021 with no results. No qualified systematic reviews, as defined by the NNR2023 project, were available.

## Meal patterns in Nordic and Baltic countries

Meal patterns have been assessed in national surveys in Finland, Iceland, Norway, and Sweden (11,12,13,14,15,16). In Finnish preschool children aged 3–6 years (*N* = 557), 88–100% of the days recorded (1–4 days) included breakfast, lunch, afternoon snack, dinner, and evening snack; of which, the lunch and dinner meals contributed most to the total energy intake (11). Regular breakfast eating was also reported among 6-year-old Icelandic children (*N* = 4,360) (12). UNGKOST 2015 in Norwegian children and adolescence aged 9 and 13 years showed that ≥90% of the 9 years old children ate breakfast, lunch, and dinner every day. Among the 13-year-old adolescents, daily eating of breakfast, lunch, and dinner was reported by 81, 71, and 92%, respectively. A majority of all participants did also have an evening eating occasion. Among the teenagers, girls were skipping meals more often than boys (13). Among Swedish adolescents (*N* = 3,099, aged 11–18 years), it was shown that about 22% of energy intake was in-between meals (14).

Among Norwegian adults in Norkost 3, meal patterns were assessed by two 24-h recalls and showed that on both days, 92% of participants consumed breakfast, 59% of participants consumed lunch, 89% of participants consumed dinner, and 33% of participants consumed an evening meal. The mean number of snacks including drinks (all eating or drinking occasions ≥50 kJ) was 1.6 per day for men and 1.9 per day for women. Moreover, 93% of men and 97% of women consumed at least one snack. In the meal pattern with three main meals, the snacks contributed to 29% of energy intake in men and 31% in women (15). In the FinDiet 2017 study, it was shown that the number of eating or drinking occasions was, on average, seven times per day, and almost all participants ate breakfast. The main meals contributed to 60% of the daily total energy intake, and most of the energy was eaten at lunch time 11–12 o´clock and at dinner about 17 o´clock (16). In addition to the national dietary surveys cited above, meal patterns were assessed in a cross-sectional, questionnaire-based internet survey with stratified random samples of Danish, Finnish, Norwegian, and Swedish adults in the age of 15–80 years (*N* = 7,531, completion rate 9–13%). This survey showed that most of the respondents in all the Nordic countries reported an eating frequency of 4–5 or more meals (not defined) per day. A regular eating pattern was seen with the majority eating breakfast, lunch, and dinner (17).

## Meal patterns and health outcomes relevant for Nordic and Baltic countries

In Nordic and Baltic countries, about 17–44% of total deaths are caused by cardiovascular diseases among all ages. High blood pressure, low density lipoprotein (LDL) cholesterol, blood glucose, and high BMI are important risk factors for cardiovascular diseases in men and women (18). Furthermore, metabolic syndrome, that is abdominal obesity, increased blood pressure, impaired glucose metabolism, and/or dyslipidemia, increases risk for cardiovascular diseases (19).

## Evidence for setting guidelines for meal patterns on health-related outcomes

We categorized meal patterns into eating frequency, snacking, skipping breakfast, timing, and intermittent fasting. Most of the reviews included cross-sectional studies, not relevant for setting guidelines. Therefore, we focused on the prospective studies and randomized clinical trials (RCTs) included in the reviews when referring the evidence below.

### Eating frequency

At total of seven reviews (20–26) were identified (AMSTAR 2: critically low for all), and no consistent findings with regard to eating frequency on body weight and body composition were found (Table 2). Among children and adolescents, mixed results were seen in the prospective studies included in the reviews (20, 24, 25). However, a meta-analysis of 11 studies (*N* = 18,849 boys and girls, aged 2–19 years) showed that the highest category of eating frequency compared with the lowest was associated with a beneficial effect on body weight, especially in boys (not significant in girls when analyses were divided by gender), but the included studies were mostly cross-sectional, not relevant for setting guidelines (26). In adults, the reviews that included prospective studies (20, 21, 24, 25) and RCTs (20, 22, 23) showed no consistent findings, with almost equal associations in different directions. For instance, in the review by Kant, it was reported from a prospective study (*n* > 7,000 men and women) followed for 10 years that eating frequency had no detrimental effect on body weight when controlling for energy intake, but in another prospective study (*n* > 20,000 men), also followed for 10 years, a higher risk of weight gain with higher eating frequency (*P* = 0.001) was seen when energy intake was not adjusted for (24). An important notion with regard to some of the RCTs included in the reviews is about the eating frequencies that were compared and the duration of the studies. For instance, in the review by Garcidueñas-Fimbres (20), two of the six RCTs compared two low eating frequencies (one meal eaten during early evening versus three main meals per day), and the duration time of the RCTs ranged from 12 h to 18 weeks (20). Cardiovascular risk factors were reported in three reviews (20, 22, 25). Results from RCTs showed beneficial effects on glucose (20), and single arm CTs showed some beneficial associations in total- and LDL-cholesterol (22) with higher eating frequency (20). No association between eating frequency and metabolic syndrome was reported either in children (20) or adults (25). However, due to the overall critically low quality of the reviews, there is no evidence for setting a general guideline for eating frequency according to health-related outcomes.

**Table 2:** Summary of reviews on eating frequency

Reference	No. of publications	Study design	Population Number of participants/Attrition rate/dropout rate Characteristics	Intervention/Exposure	Comparator	Assessment of meal frequency	Duration/Follow up	Outcome: BW and body composition (FM, FFM)	Outcome: Cardiovascular risk factors	AMSTAR 2
Garcidueñas-Fimbres, Spain, 2021 (20)	35 publications.	9 cross sectional and 3 prospective studies in children and 15 cross sectional, 2 prospective, and 6 RCTs in adults (4 cross-over and 2 parallel group studies).	Children: *n* = 36,044 (cross sectional) and *n* = 5,118 (prospective). Age 1–10 years. Adults: *n* = 77,039 (cross sectional), *n* = 52,784 (prospective) and *n* = 313 (RCTs). Attrition rate/dropout rate NR.	Eating ≤ 6 times/day	Eating > 6 times/day	Self-report, energy percentage contribution (% energy) or based on the hour (clock time; 4 publications).	3.5–10 years in children and 6–7 years in adults. RCTs 3 days–4.5 months.	Children: Cross sectional studies; inverse association between eating frequency and BMI, BMI *z*-score or BW in 7 publications and positive associations in 3 publications. Mixed results in prospective studies. Adults: Mixed results in cross sectional, prospective studies, and interventional studies. (almost equal associations in different directions).	Cross sectional studies in children: Met components were assessed in 4 publications with mainly no associations. Eating frequency were inversely associated with lower risk of hypertension in adults with abdominal obesity. In interventional trials, a greater eating frequency was associated with lower fasting plasma glucose (2 publications).	Critically low
Canuto, Brazil, 2017 (21)	31 publications. 1960–2016	2 prospective and 29 cross-sectional studies.	*N* = 136,052 men and women. Attrition rate NR. Aged 18–90 years. From US and Europe.	Number of meals classified as a continuous variable, 1–5 meals	Number of meals classified as a continuous variable, ≥3 meals-10	1–15 × 24 h recall, simple questions, meal pattern questionnaires, 1–7 d food record, meal pattern history	8–10 years (prospective)	14 studies reported an inverse association between eating frequency and body weight or body composition, 7 studies (1 prospective) found a positive association (NB! The results of this study are not shown, 10 studies no associations (1 prospective).	NR	Critically low
St-Onge, US, 2017 (22)	9 publications.	CT, RCT (cross over).	*N* = 19. Dropout rate not reported. Adults aged 18–51 years. Participants with normal weight, OW, and OB	All CTs were presented as single arms, the RCT cross over from 1 meal to 3 meals (1 publication).	No energy restrictions diets with meal frequency 1, 3, 6, 9, 12, 17 meals/day	NR	2–8 weeks	No significant difference in BW (9 publications)	Beneficial reduction in TC and LDL-c, with >6 meals/day (5 publications). TC, LDL-c, HDL-c, and BP increased with 1 meal pattern and TC and BP were reduced with 3 meal patterns (1 study). No effect on glucose or insulin.	Critically low
Schoenfeld, US, 2015 (23)	15 publications.	RCT, parallel and cross-over.	Number of participants NR, dropout rate NR. Young adults (18–49 years) and old adults (>50 years). Participants were lean or with OV, OB.	1–4 meals	3–9 meals or grazing	NR	2–52 weeks	BM assessed in 15 studies, FM in 10 studies, FFM in 9 studies. Meta-analysis showed NS difference in forest plot of all included studies in BM, FM, and FFM. In regression models controlling for energy intake, initial weight and weeks, NS difference between lower and higher meal frequencies for BM change.	NR	Critically low
Kant, US, 2014 (24)	16 publications	4 prospective, 2 CT, and 10 RCTs.	*N* = 30,086 (*n* = 2,473 in children and *n* = 402 in the trials). Age 8–12 years in children (girls only) and 18–75 years in adults with normal weight, OW, and OB. Attrition rate 11–50% (reported in 4 RCTs).	Lower frequency of eating episodes, lower categories of eating occasions (prospective). Lower than 3 eating episodes in trials.	Higher frequency of eating episodes, higher categories of eating occasions (prospective). 3–9 eating episodes or eating a minimum of grazing; 100 kcal every 2–3 h (trials).	24 h recall at baseline, predefined questionnaires 8 categories, 3–7 days diet record (prospective). NR in trials.	Prospective 6–10 years. Trials 6 days – 52 weeks.	Prospective studies in girls, the study including (*n* > 2,000) participant. Showed inverse association of eating frequency ≥6 /d at baseline with BMI change at follow-up *P* = 0.013, controlling for multiple confounders, including energy intake and dieting. In the other prospective study (*n* = 101 girls), a smaller increase in BMI *Z*-score with 4–5.9 episodes relative to ≥6, frequency of 0–3.9 occasions did not differ from ≥6. Adults: NS in general population when controlling for energy intake, and higher risk of weight gain with higher eating frequency *P* = 0.001 in men (health professionals) when no adjustment for energy intake. NS reported in 11 trials. NS in 5 RCTs in free living, hypo-energetic diets for weight loss.	NR	Critically low
Mesas, Spain, 2012 (25)	39 publications.	31 cross-sectional (10 studies adjusted for confounders), 8 prospective (3 studies adjusted for confounders).	*N* = 118,762. Attrition rate 42–97%, NR in 46%. Age 1–19 years and 20–91 years. Participants with normal, OW, and OB	≤2, 3, 4 meals per day, range 1–12 or inter quartile range	≥3 meals per day, range 1–12 or interquartile range	FFQ, 3-days food diary, 2 × 24 h recall (3 publications) otherwise simple questionnaires	1–10 years	In children: 14 cross sectional studies and 6 prospective studies showed mixed results. In adults: 17 cross and 2 prospective studies with mixed results.	No association between eating frequency and metabolic syndrome in adults (1 publication)	Critically low
Kaisari, Greece, 2011 (26)	21 publications from 11 studies.	10 cross-sectional studies and 1 case-control study	*N* = 18,849 participants. Attrition rate NR. Age 2–19 years.	Lower EF	Higher EF	FFQ (2 publications), 24-h dietary recalls (2 publications) and self-reported questionnaires completed by children (aged 10–19 years) themselves or by their caregivers/parent.		The highest category of eating frequency compared with the lowest was associated with a beneficial effect on BW (OR = 0.78, log OR = -0.24 [95% CI -0.41, -0.06]). The observed beneficial effect remained significant in boys (OR = 0.76, log OR = -0.27 [95% CI -0.47, -0.06]), but not in girls (OR = 0.96, log OR = -0.04 [95% CI -0.40, 0.32], *P* for sex differences = 0.14). Higher EF associated with lower BW in boys (8 publications), not in girls (7 publications).	NA	Critically low

AMSTAR 2 = A MeaSurement Tool to Assess systematic Reviews 2; BF = body fat; BM = body mass; BMI = body mass index; BP = blood pressure; BW = body weight; CI = confidence interval; CT = clinical trial; FFM = fat free mass; FFQ = food frequency questionnaire; EF = eating frequency; HDL-c. = high density lipoprotein cholesterol; LDL-c = low density lipoprotein cholesterol; OB = obesity; OR = odd ratio; OW = over weight; RCT = randomized clinical trial; NA = not assessed; NR = not reported; NS = non-significant; PCOS = polycystic ovarian syndrome; TC = total cholesterol; T2DM = type 2 diabetes mellitus; US = United States.

### Snacking

We identified three reviews (25, 27, 28) with AMSTAR 2 critically low for all (Table 3). Generally, in the reviews, snacking was defined as eating or drinking between meals. In children and adolescents, most of the publications included in the reviews were cross-sectional, with contradicting or null results. Mixed results were also seen in the prospective publications (25, 27, 28). Among adults, some consistency was seen as both the cross-sectional and prospective publications showed positive associations between snacking and body weight (25). None of the reviews reported cardiovascular outcomes. In most of the individual publications reported in the reviews, snacking was assessed by a simple question, ‘yes or no’, that did not indicate the number of snacks or the energy and nutrient content of the snack (Table 3). This makes interpretation difficult but indicates that among adults snacking may increase body weight. However, data on snacking quality are mostly lacking. In summary, there is no evidence for setting a general guideline about snacking according to health-related outcomes.

**Table 3:** Summary of reviews on snacking

Reference	No. of publications	Study design	Population Number of participants/dropout/retention rate, characteristics	Intervention/Exposure	Comparator	Assessment of snacking	Duration/Follow-up	Outcome BW and body composition	Outcome Cardiovascular risk factors	AMSTAR 2
Williamson, US, 2020 (27)	3 publications.	2 cross-sectional studies, 1 RCT.	*N* = 2,604. Attrition rate/dropout rate NR. Age 13–17 years with different SES.	Low frequency of snacking defined as eating foods or consuming caloric beverages between meals	High frequency of snacking defined as eating foods or consuming caloric beverages between meals	FFQ, 24-h dietary recall (self-report).	RCT 12 months	1 publication, high snacking sign. increased OW/OB (*P* < 0.028), but NS controlling for SES. 1 publication high snacking, sign. associated with OW/OB sign. in boys (*P* < 0.0001), not in girls. 1 study (RCT) NS.	NA	Critically low
Larson, US, 2013 (28)	32 publications.	2 case-control, 23 cross-sectional, 7 prospective	*N* = 92,059. Retention rate/attrition rate 70.5–99.6% NR 50%. Age 2–19 years, from US, Africa, Asia, and Europe.	Low frequency of snack consumption, percentage of energy from snacks, or the consumption of energy-dense snack foods	High frequency of snack consumption, percentage of energy from snacks, or the consumption of energy-dense snack foods	Simple questionnaires, multiple 24 h recalls, FFQ, dietary history, reported by parents and children or by the participant themself in older age groups.	NR or 3–10 years	Positive associations were seen in the 2 case-control studies. 12 publications showed no relationship, 7 showed positive associations and 4 inverse associations in the cross-sectional publications. In 6 of the prospective studies, no associations were seen, in 1 a positive association was seen.	NA	Critically low
Mesas, Spain, 2012 (25)	44 publications of whom 18 with good control of confounders (reported here).	10 cross-sectional and 7 prospective studies, 1 case-control.	*n* = 48,340 in children and adolescents age 3–19. Adults *n* = 113,813, age 20–77 years. Retention rate/50–100%, NR 41%	No snacking, low snacking defined as eating between meals, consuming small portions of food or packaged food or eating specific foods (e.g. fried and salty food, sweets, cakes), kcal/day. SF times/day, ≤1 day times/week, never, seldom, rarely	1 or more snack, high snacking defined as eating between meals, consuming small portions of food or packaged food or eating specific foods (e.g. fried and salty food, sweets, cakes), kcal/day. SF times/day, ≥3 times/day ≥5 times/week, frequently, always, times/week	Mostly simple questionnaires (eating between meals yes/no).	Prospective studies 3–8 years	Children and adolescents: Mixed results in cross-sectional studies. In the 2 publications of prospective publications with good control for confounders, no associations were seen in 1 publication and 1 publication in Finnish twins found positive association between snacking av BMI ≥ 27. In adults, both the cross-sectional (4 studies) and prospective studies (4 studies) with good control for confounders consistently showed that eating between meals, eating snack foods and frequency of snacking episodes were associated with excess weight. Not in adults >65 years (1 study)	NA	Critically low

AMSTAR 2 = A MeaSurement Tool to Assess systematic Reviews 2; BW = body weight; FFQ = food frequency questionnaire; NA = not assessed; NR = nor reported; OB = obesity; OW = over weight; RCT = randomized clinical trial; SES = socioeconomic status; SF = snacking frequency; US = United States.

### Breakfast skipping

A total of seven reviews were identified on breakfast eating or skipping (25, 29–34) with AMSTAR 2 – low (29, 30, 33) and AMSTAR 2 – critically low (25, 31, 32, 34). Body composition and cardiovascular risk factors were assessed in a minority of publications included in the reviews (Table 4). In children and adolescents, the findings were mixed in the prospective publications reported, but the reviews showed some consistency in cross-sectional publications in that skipping breakfast was associated with increased prevalence of overweight and obesity. However, as noted earlier, cross-sectional studies are not relevant for setting guidelines. In adults, meta-analyses (including prospective studies) showed skipping breakfast to be associated with about 20% increased risk for heart disease (31) and type 2 diabetes mellitus (34). No definition of the breakfast according to time of the day or nutrient content was reported. Hence, given the overall critically low quality of the reviews, these findings have to be interpreted with caution. It is difficult to know from the included publications how often breakfast has to be consumed to be preventive, and information of breakfast quality or timing is lacking.

**Table 4:** Summary of reviews on breakfast skipping

Reference	No. of publications	Study design	Population: Number of participants. Attrition rate/dropout rate. Characteristics.	Intervention/Exposure	Comparator	Dietary assessment methods	Duration/Follow-up	Outcome: BW and body composition (FM, FFM)	Outcome: Cardiovascular risk factors	AMSTAR 2
Ricotti, Italy, 2021 (29)	16 publications.	11 RCTs and 5 prospective.	*N* = 50,066. Age 1–20 years.	Interventions programs for eating breakfast (definition of breakfast varied between studies)	No intervention programs for eating breakfast	FFQ, recall-based methodology, food diaries, simple questionnaires (yes/no)	RCT 2–8 months or prospective 1–6 years or NR (1 publication)	The breakfast eating programs were effective in most of the studies. BW reported in 2 prospective and 3 RCTs. RCTs NS on BW. In the 2 prospective publications, participants with OW/OB were more likely than normal-weight participants to skip breakfast at follow-up.	NR	Low
Bonnet, US, 2020 (30)	7 publications	RCT (parallel group)	*N* = 425, mean age 35 years (range 18–65). Normal weight, people with OW, OB mean BMI 30.1 kg/m^2^.	Breakfast skipping: Extended overnight fast until 12.00, no calories consumed before 11.00, reported to center at 08.30 water and decaffeinated coffee delivered, no eating before 11.30, fasting until 10.00, instructed to eat a total of 1,200 kcal in 2 meals per day	Breakfast eating: ≥700 kcal eaten before 11.00, eating breakfast before 10.00, reported to center at 08.30 breakfast and drinks delivered, eating 15% of EI within 1.5 hours awakening/before 08.30, consuming breakfast before 09.45, instructed to eat a total of 1,200 kcal per day in 3 meals.	Self-reported and verified with continuous blood glucose monitoring, food diary, direct observation, breakfast delivered or muffins provided	4–16 weeks	Participants who skipped breakfast had a greater reduction in body weight (WMD = −0.54 kg [95% CI: −1.05, −0.03], *P* = 0.04], *I*^2^ =21.4%; *P* heterogeneity = 0.27): No effects on other body composition parameters.	2 or 3 studies reporting different parameters. LDL significantly increased by 0.24 mmol/l (95% CI: 0.06, 0.42; *P* = 0.01, *I*^2^ = 3.2%; *P* heterogeneity = 0.36) in breakfast skippers. No effects on other cardiometabolic risk parameters.	Low
Tagagi, Japan, 2019 (31)	8 publications	2 case-control, 1 cross-sectional, 5 prospective cohort studies. Included in the meta-analyses were 5 publications adjusted for confounders and 3 publications were not adjusted.	*N* = 284,484, mean age 41–71 years.	Never or seldom eating breakfast, eating breakfast <5 times/week, eating breakfast 0–2 times/week, skipping breakfast >3 times/week	Eating breakfast 2–3 times /week, most mornings, every day	NR	2–19 years or 112,000 to >1 million person years	NA	The primary meta-analysis combining HRs for Q1 (most skipping breakfast) versus Q4 (least skipping breakfast) HR/OR = 1.24 [95% CI 1.09, 1.40], *P* = 0.001	Critically low
Monzani, Italy, 2019 (32)	39 publications.	Cross sectional and prospective studies; only cross-sectional data reported.	*N* = 286,804 children and adolescents of whom 16,130 subjects were investigated for cardiometabolic outcomes. Age 44 months – 21 years.	Different definitions, mostly eating breakfast no, not every day, occasionally.	Different definitions of breakfast consumptions, mostly; eating breakfast yes, 1–7 days/week.	FFQ, validated questionnaires, 24-h recall methods, food diary, not specified	No follow-up reported.	Prevalence of skipping breakfast ranged 10–30%, with an increasing trend in adolescents, mainly in girls. Skipping breakfast was associated with OW/OB in 33 publications, of which, 2 studies in girls, NS in 5 publications).	Skipping breakfast was associated with a worse lipid profile, blood pressure levels, insulin-resistance, and metabolic syndrome.	Critically low
Sievert, Australia, 2019 (33)	13 publications. From 1990 to 2018.	RCTs (parallel groups and cross-over trials)	*N* = 1,416 of trials examining body weight *n* = 486 and energy intake (*n* = 930). Mean age 18–44 years. Normal weight, people with OW, OB.	Eating breakfast 1.5–2 h after awakening or before 11.00.	Not eating breakfast, drinking only water (matching the volume of the breakfast) before noon.	Breakfast eating directly assessed in laboratory visits, food records or recall methods.	2–16 weeks (in examination of weight change) and 2 × 8 h, 2 × 24 h and 16 weeks (energy intake).	Meta-analysis (7 trials) found a small difference in weight favoring participants who skipped breakfast (mean difference 0.44 kg [95% CI 0.07, 0.82], *I*^2^ = 43%). Meta analysis of energy intake (10 trials) showed participants assigned to breakfast had a higher total daily energy intake (mean difference 259.79 kcal/day, [95% CI 79, 441], *I*^2^ = 80%).	NA	Low
Bi, China, 2015 (34)	8 publications.	Prospective cohort (4 publications, case-control (1 publication), cross- sectional (3 publications) from Japan, China, Europe, and US).	*N* = 106,935 participants and 7,419 reported T2DM outcomes.	Never non-consumer irregular breakfast consumption 0–6 days/week	Every day, consumer regular breakfast consumption 1–7 days/week	Questionnaire, FFQ	6–18 years (prospective)	NA	A pooled adjusted RR for the association between breakfast skipping and T2DM risk was 1·21 [95% CI 1·12, 1·31], *P* = 0.984, *I*^2^ = 0.0%) in cohort studies and the pooled OR was 1·15 [95% CI, 1·05, 1·24] *P* = 0.770, *I*^2^ = 0.0%) in cross-sectional studies	Critically low
Mesas, Spain, 2012 (25)	73 publications, of whom 71 publications skipping breakfast	62 cross-sectional studies (16 adjusted for sociodemographic variables, physical activity and energy or food intake), 9 prospective studies (5 adjusted for confounders), 1 case-control (adjusted for confounders).	*N* = 364,863 (children *n* = 236,004, adults *n* = 128,859). Retention or response rate 42–98%; NR in ~ 40%. Age 2–74 years.	Skipping breakfast 1–7 days a week, always or some-times; mostly <3 times/week	Eating breakfast 1–7 days a week, always or sometimes, mostly ≥3 times/week	3–7 days food diary, simple questionnaires.	1–10 years (prospective studies)	In children/adolescents, 35 of the 48 cross sectional studies found that skipping breakfast was associated with OW/OB. In the 5 prospective studies + 1 case-control with good control for confounders, no association in 4 publications, in 2 publications, no change in weight in normal weight, but significant decrease in BMI with skipping breakfast among participants with OW/OB. In adults, 8 cross sectional studies found an association between skipping breakfast and obesity, 5 did not. In 2 of the prospective publications (men only), positive associations were found between breakfast skipping and weight gain.	In 1 cross-sectional study, no association was found between skipping breakfast and metabolic syndrome.	Critically low

AMSTAR 2 = A MeaSurement Tool to Assess systematic Reviews 2; BMI = body mass index; BW = body weight; CI = confidence interval; EI = energy intake; FFQ = food frequency questionnaire; FM = fat mass; FFM = fat free mass; HR = hazard ratio; LDL-c. = low density lipoprotein cholesterol; NA = not assessed; NR = not reported; OR = odd ratio; OB = obesity; OW = overweight; RR = risk ratio; RCT = randomized clinical trial; T2DM = Type 2 diabetes mellitus; WMD = weighted mean difference; US = United States.

With regard to body weight, in the meta-analyses by Bonnet et al. (30) and Sievert et al. (33) including only RCTs in adults (N~450 in each of the meta-analyses), the effect of eating or skipping breakfast was investigated. Both reviews, with overlapping studies included, showed skipping breakfast to be associated with about 0.5 kg weight loss in participants with normal weight, overweight, and obesity during 2-16 weeks (30, 33). The breakfasts varied among studies, but mostly participants prolonged their overnight fast and had lunch about noon in the skipping breakfast groups. Interpreting these results may indicate that delaying the first meal of the day seems not detrimental for control of body weight in adults.

Cardiovascular risk factors were reported in three reviews (25, 30, 32). Among children, cross-sectional data showed that skipping breakfast was associated with impaired effects on lipid profile, blood pressure, and insulin (32). In adults, increased LDL-cholesterol was reported (30) in RCTs, but no associations between breakfast skipping and metabolic syndrome were seen in one cross-sectional study (25).

Overall, the evidence with regard to breakfast skipping on health-related outcomes is limited, and no general guideline about skipping or eating breakfast can be set as long as energy and nutrient needs are met.

### Timing

We identified four reviews on meal timing (35–38) with AMSTAR 2 low (35, 36) and critically low (35, 36). Overall, the evidence is limited (Table 5). However, the reviews showed consistency in that lower energy intake during late afternoon or evening was related to lower body weight or weight loss (in prospective studies) (35–37). However, a meta-analysis of RCTs did not show significant associations between late eating and body weight (36). To be noted is that the RCTs included in the meta-analysis were mostly of short duration and hypo-caloric, with low generalizability for free-living conditions. Cardiovascular risk factors were reported in two reviews (35, 38), showing reduction in markers for glucose metabolism with lower energy intake later in the day (35, 38). This may indicate that a lower energy intake during the evening may be beneficial for body weight and glucose metabolism. However, the evidence of meal timing on health-related outcomes is limited, and no guideline can be set.

**Table 5:** Summary of review on timing

Reference	No. of publications	Study design	Population Number of participants/dropout rate, characteristics	Intervention/Exposure	Comparator	Assessment of dietary intake/timing	Duration/Follow-up	Outcome BW and body composition (FM, FFM)	Outcome Cardiovascular risk factors	AMSTAR 2
Becutti, Italy, 2017 (35)	10 publications	4 prospective and 6 RCTs (parallel groups or cross over).	*N* = 6,401 participants of whom (*n* = 5,488 from general population) with normal weight, OW, OB and patients (bariatric surgery, PCOS; *n* = 330), RCTs (*n* = 223).	Eating lunch after 15:00, eating the larger percentage of total energy later. 2 RCTs acute consumption of meals 18.00 and 23.00.	Eating lunch before 15:00, eating the larger percentage of total energy early. 2 RCTs acute consumption of meals 08.00 and 18.00.	24 h recall, food record (reported in 2 prospective publications). NR in 6 relevant publications.	Prospective 1–7 years. RCTs acute consumption, 2–12 week.	In the 2 prospective studies with moderate risk of bias, 1 showed improved weight loss after 20 weeks of intervention in early lunch eaters. The other study showed increased incidence rate of obesity and T2DM from the lowest to the highest energy intake at dinner. In a multiple regression model, risk for obesity increased by late eating (OR = 2.33; 95% CI [1.17, 4.65], *P* = 0.02). 2 RCTs reported BW. 1 study showed improved weight loss when higher amount of energy was eaten at breakfast compared to dinner in a hypocaloric diet among women with OW/OB. The other study among normal weight women showed no difference between energy consumed at breakfast or dinner during weight maintenance.	Sign. increased markers for glucose metabolism i.e. glucose, interstitial glucose (1 publication), insulin, HOMA-IR after higher energy intake later in the day in 6 RCTs.	Low
Fong, Australia, 2017 (36)	18 publications, of which 9 publications were included in the meta-analysis.	4 cross sectional studies and 5 CTs.	*N* = 7,058, of which *n* = 6,685 cross citational and CTs *n* = 370 (4 studies only in women), mean age 29–64 years with normal weight, OW, OB in cross sectional studies. OW, OB in CTs.	Cross sectional: Evening energy intake from 17, 18, and 19 until midnight CT: Hypo caloric (weight loss) diet with either 1 single meal (~600–700 kcal) eaten in the evening or most of the energy intake was eaten at dinner/evening meals.	Cross sectional: Daytime energy intake before 17, 19, and 20 CT: Hypocaloric (weight loss) diet with 1 single meal eaten in the morning or hypocaloric meals were distributed with higher energy intake before the dinner/evening meals.	Cross sectional: single question, 24-h recall. CTs: 3–7 days food diary checklists reviewed by dietitian, 3-day food diary, feedback form reporting on compliance.	18 days – 32 weeks in CTs.	Meta-analysis cross sectional studies showed NS trend between lower BMI and lower evening intake – 0.39 kg/m^2^ [95% CI −0.80, 0.01], *P* = 0.06). The meta-analysis of CTs showed no difference in weight change between small and large dinner groups eating hypocaloric diets (−0.89 kg; [95% CI −2.52, 0.75], *P* = 0.29).	NR	Low
Almoosawi, UK, 2016 (37)	10 publications	8 cross-sectional and 2 prospective.	*N* = 13,300 (*n* = 11,311 cross-sectional, *n* = 1,589 prospective studies). Attrition rate NR. Men/Women. Age range 3–91 years. General population.	Evening-to-morning energy intake ratio >2, energy intake at supper, energy intake in the evening ≥33%.	Morning intake, energy intake early.	1–4 days of 24 h recall, 3–8 dietary records, diet history.	3–7 years in girls (1 study) and 10 years in adults (1 study)	In 6 cross sectional studies, mixed results between BMI and late eating were shown, positive associations shown in 2 studies. In prospective study in girls, the evening intake was positively associated with increased BMI z score at follow-up. 1 prospective study in adult men did not report on BW.		Critically low
Varady, US, 2016 (38)	1 publication; during 2015–2016.	RCT, cross-over, 1 week wash-out.	*N* = 32, 24 years, normal weight, women	3 meals per day, lunch at 4.30 pm, maintenance diet	3 meals per day, lunch at 1.30 pm, maintenance diet	Meals provided	7 weeks	NS difference between groups.	Glucose and HbA1c AUC ↑after late lunch	Critically low

AMSTAR 2 = A MeaSurement Tool to Assess systematic Reviews 2; AUC = area under the curve; BMI = body mass index; BW = body weight; CI = confidence interval; CT = clinical trial; FM = fat mass; FFM = fat free mass; HbA_1_c = glycated hemoglobin A_1_c; HOMA-IR = homeostasis-model assessment insulin resistance; NW = normal weight; OW = over weight; OB = obesity; OR = odd ratio; PCOS= polycystic ovarian syndrome; RCT = randomized clinical trial; T2DM = Type 2 diabetes mellitus; UK; United Kingdom; US = United States.

### Intermittent fasting

We identified six reviews that included participants with normal weight, overweight, and obesity (22, 39–43), AMSTAR 2 – critically low for all. Generally, the reviews included studies with different variants of intermittent fasting (Table 1) and showed that intermittent fasting reduced body weight (Table 6). The reduction in body weight and fat mass was about 3–14% during 3–14 weeks (22, 43) but not superior to continuous energy restrictions. Importantly, the reviews included publications not comparing similar energy restrictions between intermittent fasting and continuous energy restrictions. Limited and mixed results were seen with regard to effects on fat free mass. Cardiovascular risk factors were reported in four reviews (22, 39, 40, 43). Some beneficial effects were seen in triglycerides, high density lipoprotein (HDL) cholesterol, and glucose (22, 43), as is expected due to weight reduction. Recent reviews (not from search) including only participants with overweight or obesity (44, 45), with AMSTAR 2 critically low (44) and low (45), respectively, showed the intermittent fasting diet to improve body weight and lipid profile during 1 year of follow-up (44, 45). However, the meta-analysis by He et al. (45), including 11 RCTs (*N* = 850 participants) with similar energy restrictions between the intermittent or continuous energy restricted groups, showed a marginally greater weight loss (weight mean difference: −0.95 kg [95% CI: −1.63, −0.27], *P* = 0.01) in the intermittent groups. Subgroup analysis showed this to be driven by short time studies (*n* = 300) with duration of 2–3 months (weight mean difference: −1.66 kg [−2.44, −0.27], *P* = 0.0001), with no difference between groups in longer term studies (*n* = 550) of 6–12 months (weight mean difference: −0.09 kg [−1.05, 0.87] *P* = 0.85). No significant findings in waist circumference, blood lipids, blood pressure, glucose, or HbA1c were seen (45). A review by Guerrero et al. including 17 RCTs (*N* = 1,091 participants) showed good adherence that may decline over time (reported in about 60% of the included studies) (44).

**Table 6:** Summary of reviews on intermittent fasting

Reference	No. of publications	Study design	Population Number of participants/dropout rate, characteristics	Intervention/Exposure	Comparator	Duration/Follow-up	Outcome BW and WC	Outcome Body composition (FM, FFM/LBM)	Outcome Lipids (TC, LDL-c., HDL-c., TG)	Outcome Blood pressure (SBP, DBP) Glucose, Insulin, IR	AMSTAR 2
He, China, 2021 (45)	11 publications	RCT	*N* = 987 included (W: 712, M: 275). Mean age range 39–68 years. *N* = 850 completed and including in the meta-analysis. Dropout rate 14% similar between groups. Overweight or obesity.	IER diet (2–3 days/week) 3 studies ADF and 8 studies 5:2.	CER diet with similar energy restrictions as IER diet	Duration time 2–12 months/ follow up 1–12 months.	Meta analysis showed absolute weight loss (WMD: −0.95 kg; 95% CI: −1.63 to −0.27; *P* = 0.01) in favor of IER. NS difference between groups for WC (4 publications).	Sensitivity analyses showed NS between group differences in % FM or % FFM (8 publications).	Most studies showed NS between group effects on lipids (8 publications), BP (6 publications).	Sensitivity analysis showed a significant reduction in fasting insulin (WMD: −1.14 μU/mL; 95% CI: −1.81 to −0.47; *P* = 0.0008) and HOMA-IR (WMD: −0.22 mmol/L × µU/mL; 95% CI: −0.40 to −0.04; *P* = 0.02) with IER (4 publications). NS i fasting glucose (8 publications).	Low
Guerrero, Spain, 2021 (44)	18 publications (17 studies)	RCT	*N* = 1,331 included/*N* = 1,091 completers. Dropout rate~ 18%. M/W, 18–70 years, BMI > 25 kg/m^2^	IF diet (ADF, 5:2, 4:3). In 3 publications, CER was also included as an intervention.	CER diet, regular diet (2 publications), according to energy needs (1 publication).	Duration time 6–48 weeks/follow-up 4–52 weeks (7 publications with no follow up)	Reported in 17 publications. 13 publications showed NS difference in WL between IF and CER diets. WC was assessed in 9 publications, of which 8 publications NS.	Reported in 13 publications. 8 publications showed NS difference in body composition between IF and CER diets.	Reported in 13 articles. 10 articles showed NS difference in any of the lipid profile variables between IF and CER diets.	NR.	Critically low
Gabel, US, 2021 (39)	13 publications, of which 12 trials.	8 RCTs (parallel groups and cross-over), 1 RT (2 cross-over; 2 treatment groups), 2 CT single arm, 1 CT parallel groups.	*N* = 456 men and women with normal weight, OW, OB. Age NR (adults). Dropout rate NR.	4–10 h TRE, self-selected or described, time restricted eating from 08 to 17 compared to later eating window (1 RT)	No meal timing restrictions, TRE from 12 to 21 compared to earlier eating window (1 RT).	1–16 weeks	↓BW in time restricted feeding group (4–8 h) compared to control group- BW decreased 2–4% in 3 RCTs in participant with OB. In normal weight, NS in 2 RCT and 2% BW ↑ in time restricted eating group + resistant exercise (1 RCT).	↓FM in 2 RCTs and ↓ LBM 3 RCTs in obese. ↓FM and ↑LMB in normal weight in time restricted eating group + resistant training group (1 RCT). NR FM 5 trials and NR lean mass 6 trials.	9 trials reported lipids between groups. NS difference in TG between groups in 7 trials. NS differences in LDL or HDL.	9 trials reported BP between groups. NS in 7 trials and ↓BP in 2 trials	Critically low
Stockman, US, 2018 (40)	14 publications (13 studies)	RCT	*N* = 707/dropout rate 0–40% (not reported in 3 publications). Men/women, 18–70 years, BMI >18.5 kg/m^2^	ICR, ADF, Muslim Sunnah fasting with counseling	CER diet, regular diet, exercise (1 publications)	8 days – 6 months	Reported in 13 publications IF: WL ↓ 2.5–9.9%, mixed results when comparing IF with CER.	Reported in 10 publications. ↓ FM associated with WL. FFM reported in 4 publications, NS in 2 publications, ↓ FFM 2 publications	Reported in 5 publications. Mixed results depending of the comparator.	Reported in 3 publications. Mixed results depending on the comparator.	Critically low
Patterson, US, 2017 (41)	16 publications (14 studies)	CT, RCT (parallel group and cross-over)	*N* = 558/dropout rate NR. Men/women, young to middle aged adults with normal weight, OW, OB.	ADF, MFR, TRF (1 meal per day, prolonged fasting ≥11 h/day, skipping breakfast)	No control group, usual eating habits, CER, TRF (3 meals per day, usually fasting interval, including breakfast)	1 day – 6 months	Reported in 15 publications. ADF (3 articles, 1–22 days in healthy non-obese) WL mixed results. MFR WL ↓ 3.2–8.0% in 7 of 9 publications. TRF WL (in 2 of 3 publications, WL not assessed in 1 article)	NR	Reported in 12 publications. ADF (1 article) ↑LDL, ↑HDL, ↓TG. MFR (8 publications) mostly NS changes in lipids. TFR mixed results (3 articles)	Reported in 12 publications. ADF ↓ gluko-regulatory markers. MFR mixed results in glucose and insulin. TRF (3 publications) mixed results.	Critically low
St-Onge, US, 2017 (22)	12 publications (of which, 2 publications from observational studies)	CT, RCT, prospective.	*N* = 334 dropout rate NR. Men/women, 18–75 years, with normal weight, OW, pre-diabetes, OB.	ADF, PF	Comparator NR	3–24 weeks	Reported in 10 publications. ↓BW ~ 3–14%	NR	Reported in 10 publications. Variable results on total TC and LDL-c. depending on baseline levels. TG↓ dependent on WL. Mixed results HDL-c. 1 observational study reported lower risk of CAD among cardiac catheterization patients.	BP and glucose regulatory markers reported in 8 publications. ↓SBP and DBP in studies with 6–7% WL. ↓ IR in studies with 4% WL. 1 observational study reported lower risk of T2DM among cardiac catheterization patients.	Critically low
Horne, US, 2015 (42)	7 publications	3 RCT and 2 prospective studies	*N* = 796 (of which *n* = 151 in RCT studies)/dropout rate NR. Participants with normal weight, OW, OB.	ADF, IF, WDF in RCT. 1 publication 24 h water only. Fasting in the prospective publications were primarily religious, usually once/month.	RCT non-intervention or standard diet control group.	RCT 2 days – 12 weeks. Observational studies decades.	WL reported in all 3 RCT publications	1 RCT reported changes in FM	1 RCT reported changes in total-c., LDL-c. 1 observational study reported lower risk of CAD (adjusted OR = 0.46 [95% CI 0.27, 0.81], *P* = 0.007) among cardiac catheterization patients.	1 RCT reported changes in BP. 1 observational study reported lower risk of T2DM (adjusted OR = 0.40 [95% CI 0.16, 0.99], *P* = 0.044) among cardiac catheterization patients.	Critically low
Tinsley, US, 2015 (43)	20 publications (15 studies)	CT, RCT	*N* = 587/dropout rate NR. Men/women, 33–60 years with normal weight, OW, OB.	ADF, WDF, TRF	No control group, control group without energy restriction, control group with CER or exercise.	ADF 3–12 weeks, WDF 12–24 weeks, TRF 16 weeks	Reported in 20 publications. ADF ↓BW ~ 3–7%, WDF ↓BW ~ 3–9%, TRF (1 article) BW NR.	Reported in 20 publications. ADF ↓BF ~ 3–5.5 kg. ↓FFM (reported in 4)	Reported in 11 publications. ADF ↓TC ~10–21% and ↓TG ~14–42%, WDF ↓TC ~ 5–20% and ↓TG ~17–50%	BP reported in 8 publications, mixed results. Glucose regulatory markers NR.	Critically low

ADF = alternate day fasting; AMSTAR 2 = A MeaSurement Tool to Assess systematic Reviews 2; BMI = body mass index; BP = blood pressure; CAD = coronary artery disease; CER = continuous energy restriction; CR = calorie restriction; CT = clinical trials; CVD = cardiovascular disease; DBP = diastolic blood pressure; FM = fat mass; FFM = fat free mass; HDL-c. = high density lipoprotein cholesterol; ICR = Intermittent calorie restrictions; IER = intermittent energy restrictions; IF = intermittent fasting; IR = insulin resistance; LBM = lean body mass; LDL-c. = low density lipoprotein cholesterol; OB = obesity; OR = odd ratio; OW = over weight; PF = periodic fasting; RCT = randomized clinical trials; MFR = modified fasting regimens; NR = not reported; NS = non-significant; SBP = systolic blood pressure; TC = total cholesterol; TG = triglycerides; TRE = time restricted eating; TRF = time restricted feeding; T2DM = type 2 diabetes mellitus; WC = waist circumference; WDF = whole day fasting; WL = weight loss; WMD = weight mean differences.

↓ = significant reduction compared to control group; ↑ = significant increase compared to control group.

To summarize, the evidence is limited, and no guideline about intermittent fasting on health-related outcomes can be set for the general population. However, among people living with overweight and obesity, intermittent energy restriction seems to be equal to continuous energy restriction for medical treatment.

## Mechanisms

The human night and day cycle is of importance for timing of meals. Lately, the impact of circadian rhythm and chrono-nutrition on health has been discussed. Circadian rhythms are biological rhythms that follow a 24-h cycle, including nutritional and metabolic processes. This suggests that we may consider not only what we eat but also when we eat, as the timing of energy intake could affect chronic disease risk (46, 47).

Meal patterns may influence homeostatic regulation for body weight, glucose, and lipid metabolism through how eating frequency, regularity, and timing are synchronized according to circadian rhythms. Chrono-nutrition is an emerging field of research involving meal patterns, circadian rhythms, body weight regulation, and metabolic health (7). Circadian rhythms promote synchronicity of biological processes during approximately 24 h, and, among others, these rhythms signal sleep and awakening, feeding, and fasting (48).

The endogenous circadian system is primarily controlled by an autonomous master clock in the suprachiasmatic nucleus (SCN) of the hypothalamus, which is synchronized by light and entrains secondary clocks in the brain and most peripheral tissues of the body. These secondary clocks are also entrained by environmental cues and behaviors, such as eating and sleeping (6). When these behaviors fail to align with the master clock (SCN), mismatch may occur. This is most markedly seen among shift-workers (49). However, variability in sleep and eating pattern during the week, called social and eating jetlag (i.e. differences in time of sleeping and eating on free days and workdays) (50), may also have an influence on the synchrony of the biological processes related to body weight control and metabolism (7, 49, 51). Research on circadian rhythms in lipid and glucose metabolism and insulin sensitivity may support eating more of the total energy intake earlier in the day (7).

A factor that also may influence the effects of late eating is chrono-type (52), a measure of the individual preferences for morning or evening as can be influenced by genetic and environmental factors (50). Cross sectional studies have shown that chrono-types vary by age, with more morning types in young childhood (52), gradually more evening type during adolescence (53), and back to more morning type during older ages (54). However, this is not confirmed in prospective research but may indicate that differences in chrono-types (i.e. morning or evening) could be of importance to consider regarding the effects of meal patterns on health.

## Data gaps for future research

Different meal patterns may be associated with diet quality. In the present scoping review, diet quality was assessed in three reviews (20, 29, 32). In children and adolescence, some beneficial effect of intervention programs for eating breakfast was seen (29), but otherwise, the evidence was divergent. Among adults, meal frequency was positively associated with diet quality. This was not seen in a review by Leech et al. (55), who found a consistent inverse association between skipping breakfast and diet quality, but no associations between other meal patterns. Thus, the evidence with regard to the effect of meal patterns on diet quality is inconclusive and needs further investigation.

Research is needed to investigate the influence of meal patterns, especially with regard to meal timing, including chrono-nutrition, on body weight and composition, metabolism, and cardiovascular disease risk factors in all age groups. In this regard, more research is needed on the influence of eating breakfast on cardiovascular health. Results from the National Health and Nutrition Examination Survey III (56) confirmed the findings from the meta-analysis by Takagi et al. (31), but causality has not yet been proven. Thus, eating breakfast may be an epiphenomenon linked to dietary intake and other lifestyle factors (i.e. sleep and physical activity) (57). Moreover, research on how intake at one eating occasion influences the next should also be considered. Well-designed prospective and RCTs are needed, also including statistical analyses taking the meal patterns of the whole day into consideration with regard to energy and nutrient intake. To better understand the impact of meal patterns on health, especially body weight and metabolism in the light of meal timing, these issues need to be addressed in national surveys. Focus on how eating frequency, snacking, and timing contribute to energy intake and body weight in all ages, from children to elderly, during pregnancy and lactation, as well as metabolic risk factors should be emphasized in other well-designed trials. In addition, more research is needed on how culture and socioeconomic factors, gender, age, and different kind of households are associated with meal patterns and health. In order to reduce methodological problems regarding meal patterns, researchers ought to describe their definitions and classifications adequately. Different definitions also exist in the Nordic dietary surveys. A starting point could be to standardize this in the Nordic and Baltic countries. Consensus on the definitions of various eating occasions is crucial to make reliable comparisons and conclusions.

With regard to intermittent fasting, a higher loss of lean mass during intermittent fasting compared to continuous energy restrictions has been reported (45, 58). A lot of variants for intermittent fasting exists, and less is known about the dietary quality during fasting days. However, lower dietary quality during intermittent fasting compared to continuous energy restriction has been reported (59). Further research is needed on intermittent fasting to clarify the effect of diet quality and if lean mass/muscle mass can be preserved by optimal intake of protein including plant-based protein and physical activity.

## Limitations

All the reviews got AMSTAR 2 in the lowest categories. This was mainly due to inadequacy of the literature search (~2/3 of the reviews), risk of bias in the individual studies included (~½ of the reviews) and not considering bias when interpreting the results (~2/3 of the reviews). In addition, none of the reviews presented a list of excluded studies. Therefore, the reviews cannot provide an accurate and comprehensive summary of the available evidence. Furthermore, we selected reviews including healthy participant with normal weight, overweight, and obesity in all ages. However, meal patterns in the elderly were only reported in a minority of the reviews, and it was not possible to draw any conclusions. In addition, our search did not identify reviews of regularity other than skipping breakfast (i.e. no studies on time between meals or day-to-day variation). Moreover, due to the methods of using reviews, we may have missed newer studies not included in reviews.

## Conclusion

No causal relationship between meal patterns including intermittent fasting and health-related outcomes was found. The evidence is limited and inconclusive due to low to critically low quality of the reviews, including mostly cross-sectional studies. The research on meal patterns lacks consistency mainly because of no common definitions of meals and snacks or not taking energy intake into consideration. Thus, with the evidence at this time point, we cannot give any advice on meal patterns based on health outcomes. In this regard, meal patterns may vary within the context of an energy balanced and nutritionally adequate diet.
